# P27 A challenging case of MDR TB infection following a holiday in India

**DOI:** 10.1093/jacamr/dlad143.031

**Published:** 2024-01-03

**Authors:** Jemima Horsley Downie, Catriona Macrae, Nicholas Kennedy

**Affiliations:** NHS Lanarkshire; NHS Lanarkshire; NHS Lanarkshire

## Abstract

**Background:**

TB is an infection caused by *Mycobacterium tuberculosis*. MDR TB is caused by strains of *M. tuberculosis* resistant to isoniazid and rifampicin.^1^ XDR TB refers to strains resistant to one or more second-line injectable drugs and any fluoroquinolone, whereas pre-XDR TB refers to strains resistant to either a second-line injectable drug or a fluoroquinolone.^1^ The implications of MDR TB include prolonged treatment requirements, increased morbidity and mortality, and risk of onward transmission to others.^2^

**Case history:**

A woman in her forties with poorly controlled diabetes was admitted to hospital three times in 3 months with respiratory symptoms and pulmonary consolidation. She was treated for recurrent community acquired pneumonia (CAP). On her fourth admission, sputum and stool microscopy detected acid fast bacilli (AFB) and PCR using Xpert MTB/RIF (Cepheid® GeneXpert® systems) confirmed *M. tuberculosis* with resistance to rifampicin. She had no known TB contacts but had had a 5 week holiday in Goa, India, 2 years prior.

**Management:**

Phenotypic testing confirmed the *M. tuberculosis* isolate was resistant to rifampicin, isoniazid and aminoglycosides but susceptible to fluoroquinolones. Following discussion with the British Thoracic Society (BTS) MDR TB advice service she was empirically initiated on linezolid 600 mg BD, bedaquiline 200 mg OD, levofloxacin 1000 mg OD, clofazimine 200 mg OD, cycloserine 250 mg BD, pyrazinamide 2000mg OD, ethambutol 1000 mg OD and pyridoxine 10 mg OD. The isolate was later identified as of New Delhi Central Asia lineage, with additional resistance to pyrazinamide (which was then stopped). This is the first case of this lineage reported in the UK. Therapeutic drug monitoring of linezolid, levofloxacin and cycloserine was performed. Cycloserine was changed to terizidone 250 mg BD after 1 month, due to a national supply issue. She suffered from medication side effects including diarrhoea, nausea, derangement in liver functions tests, worsening of pre-existing peripheral neuropathy, myalgia, hypercalcaemia, itch, renal tubular acidosis and acute kidney injury. These were managed pharmacologically and through cessation of terizidone after 2 months and dose reduction, then cessation, of linezolid after 4 months.

**Outcomes:**

Clinically she improved and repeat imaging showed gradual resolution of consolidation and C-reactive protein levels decreased. She is 10 months into an anticipated 18–24 month treatment regimen, recommended due to her extensive pulmonary disease. Medication side effects are currently well controlled. The infection prevention and control team screened all contacts from this and prior admissions, with no further cases identified.

**Conclusions:**

This case highlights the importance of suspicion of TB in patients with chronic cough and radiological changes, particularly with risk factors for TB such as relevant travel history and diabetes. MDR TB infection has significant consequences and treatment remains challenging with the burden of multiple drugs, and their side effects, leading to further patient morbidity.
